# Clinicopathological and prognostic implications of *EGFR* mutations subtypes in Moroccan non-small cell lung cancer patients: A first report

**DOI:** 10.1371/journal.pone.0298721

**Published:** 2024-06-05

**Authors:** Sara Boukansa, Ismail Mouhrach, Fatima El Agy, Sanae El Bardai, Laila Bouguenouch, Mounia Serraj, Bouchra Amara, Yassine Ouadnouni, Mohamed Smahi, Badreeddine Alami, Nawfel Mellas, Zineb Benbrahim, Hinde El Fatemi

**Affiliations:** 1 Laboratory of Biomedical and Translational Research, Faculty of Medicine and Pharmacy, Sidi Mohamed Ben Abdellah University, Fez, Morocco; 2 Laboratory of Anatomic Pathology and Molecular Pathology, University Hospital Hassan II, Sidi Mohamed Ben Abdellah University, Fez, Morocco; 3 Unit of Medical Genetics and Oncogenetics, University Hospital Hassan II, Sidi Mohamed Ben Abdellah University, Fez, Morocco; 4 Department of Pneumology, University Hospital Hassan II, Sidi Mohamed Ben Abdellah University, Fez, Morocco; 5 Department of Thoracic Surgery, University Hospital Hassan II, Sidi Mohamed Ben Abdellah University, Fez, Morocco; 6 Department of Radiology, University Hospital Hassan II, Sidi Mohamed Ben Abdellah University, Fez, Morocco; 7 Department of Oncology, University Hospital Hassan II, Sidi Mohamed Ben Abdellah University, Fez, Morocco; Osmania University, Hyderabad, India, INDIA

## Abstract

**Background:**

Non-small cell lung cancer (NSCLC) remains a significant global health concern, with *EGFR* mutations playing a pivotal role in guiding treatment decisions. This prospective study investigated the prevalence and clinical implications of *EGFR* mutations in Moroccan NSCLC patients.

**Methods:**

A cohort of 302 NSCLC patients was analyzed for *EGFR* mutations using multiple techniques. Demographic, clinical, and pathological characteristics were assessed, and overall survival (OS) outcomes were compared among different *EGFR* mutation subtypes.

**Results:**

*EGFR* mutations were present in 23.5% of patients, with common mutations (81.69%) dominating. Common mutations showed strong associations with female gender and non-smoking status, while rare mutations were associated with a positive smoking history. Patients with *EGFR* mutations receiving tyrosine kinase inhibitors (TKIs) had significantly improved OS compared to wild-type *EGFR* patients. Notably, patients with common *EGFR* mutations had the highest OS, while those with rare mutations had a shorter survival period, albeit not statistically significant.

**Conclusion:**

This study highlights the relevance of *EGFR* mutation status in NSCLC patients, particularly in therapeutic decision-making. The association between smoking history and rare mutations suggests the need for tailored approaches. The survival advantage for patients with common *EGFR* mutations underscores the significance of personalized treatment strategies.

## Introduction

Lung cancer exerts a substantial global burden, afflicting more than 1.8 million patients annually. Among malignancies, lung cancer ranks as the second most prevalent cancer in males, following prostate cancer, and as the third most prevalent in females, trailing behind breast and colon cancer [1]. Alarming in its impact, lung cancer stands as the leading cause of cancer-related mortalities across genders [2].

The management of patients with non-small cell lung cancer (NSCLC) has experienced a transformative shift owing to the identification of activating mutations within the tyrosine kinase domain of the epidermal growth factor receptor (*EGFR*) and the subsequent advent of tyrosine kinase inhibitors (TKIs) that selectively target these mutations [3–5].

Patients harboring in-frame deletions of exon 19 and the L858R mutation, both of which are common drug-sensitive epidermal growth factor receptor mutations, have been predominantly enrolled in clinical trials evaluating *EGFR* tyrosine kinase inhibitors (*EGFR* TKIs) [6–10]. Collectively, these two mutations, commonly referred to as common EGFR mutations, constitute approximately 80–85% of all *EGFR* mutations [3, 4, 11–13]. The remaining 15–20% of *EGFR* alterations primarily consist of point mutations (e.g., G719X or L861Q) and exon 20 insertion mutations, which are categorized as rare *EGFR* mutations [12–14].

Recent investigations have underscored the considerable significance of establishing a potential correlation between infrequent epidermal growth factor receptor mutations and their distinctive clinicopathological characteristics, therapeutic outcomes, and subsequent impact on patient prognoses [15–17]. Notably, rare mutations such as T790M, and exon 20 insertions have been observed to be associated with both primary and acquired resistance to *EGFR* tyrosine kinase inhibitors [18–22]. Optimizing therapeutic approaches and improving patient outcomes requires a thorough understanding of the prevalence, occurrence, and consequences of these infrequent mutations.

Within the realm of thoracic oncology, a comprehensive investigation into the clinicopathological and morphological characteristics of common *EGFR* mutations assumes paramount significance. A heightened comprehension of these mutations bears substantial potential for refining diagnostic modalities, informing therapeutic decision-making, and facilitating the development of innovative treatment strategies. By concentrating on rare *EGFR* mutations, personalized medicine approaches can be optimized, ultimately leading to enhanced patient outcomes. The present study aims to assess the predictive value of *EGFR* mutation subtypes in Moroccan patients with non-small-cell lung cancer, while also comparing the clinicopathological and morphological features of common, rare, and wild-type *EGFR* mutations. Additionally, the study seeks to elucidate the prognostic implications associated with these various *EGFR* mutation subtypes.

## Patients and methods

### Study population

In the present investigation, a cohort of 302 patients diagnosed with presumed primary non-small cell lung cancer was enrolled, wherein the tumors of these patients were subjected to *EGFR* mutation testing. The study followed a prospective design, aiming to collect data in a systematic manner from 01 November 2017 to 31 May 2020. Specimens were acquired from 302 consecutive requests, originating from the medical oncology, pneumology, and radiotherapy departments, and were processed at the pathological anatomy platform situated at the Hassan II University Hospital in Fez. The ethics committee of the Faculty of Medicine and Pharmacy in Casablanca, Morocco, approved this prospective study (reference number: 17/15), and, all patients provided written informed consent. The selection criteria for participant inclusion in this study were as follows: Patients with histologically confirmed NSCLC or metastatic lung adenocarcinoma were included, along with young, non-smoking patients with metastatic squamous cell carcinoma or carcinoma not otherwise specified (NOS). Conversely, patients without histological confirmation of non-small cell lung cancer, those with insufficient tumor material, or those with incomplete medical records were excluded from the study. The clinical and anatomopathological data, including age, gender, smoking status, histological type, type of metastatic sites, stage, and survival information, were acquired from medical records encompassing anatomopathological and molecular reports.

### Molecular analysis

Tumor DNA was isolated from paraffin-embedded sections of the tumor. A pathologist identified blocks with the highest tumor cell content based on slides stained with hematoxylin, saffron, and eosin. The chosen formalin-fixed paraffin-embedded (FFPE) tumor block was utilized to obtain 4–7 sections, each measuring 5 μm in thickness. DNA extraction was performed using the QIAamp DNA FFPE Tissue Kit (Invitrogen) in strict adherence to the manufacturer’s instructions. Subsequently, the concentration of DNA (measured in ng/μl) was determined using a Qubit fluorometer.

The analysis of mutations in exons 18, 19, 20, and 21 of the *EGFR* gene was conducted, taking into consideration the availability of materials.

### Direct sequencing and real-time PCR

For the detection of *EGFR* mutations, direct sequencing and real-time PCR were performed using the Therascreen’s *EGFR* RGQ PCR kit, founded on Scorpions® and ARMS (Amplification Refractory Mutation System) technologies. These techniques were employed when the necessary materials and resources were accessible. Real-time PCR procedures followed the manufacturer’s instructions.

### Pyrosequencing

Mutation analysis was also carried out via pyrosequencing using the theraScreen® *EGFR* Pyro kit, whenever available. Pyrosequencing is known for its high sensitivity and accuracy in mutation detection. The pyrosequencing protocol was implemented according to the manufacturer’s guidelines.

### Idylla

Furthermore, the Idylla system was utilized for *EGFR* mutation analysis as materials permitted. The Idylla platform offers automated and rapid mutation detection capabilities. Analysis procedures were conducted in accordance with the Idylla protocol.

The choice of mutation analysis technique was determined by the availability of materials and resources at the time of analysis. This adaptable approach ensured that the mutation analysis was conducted with the most suitable technique based on the prevailing circumstances, while maintaining the accuracy and reliability of the results.

### *EGFR* mutation

*EGFR* mutations were systematically classified to several categories, including "Wild Type" for samples lacking *EGFR* mutations, "Common Mutation" encompassing exon 19 in-frame deletions or exon 21 L858R substitutions, "Rare Mutation" encompassed all variations distinct from these common mutations, such as G719X mutations in exon 18, S768I mutations, De Novo T790M mutations, insertions in exon 20, L861Q mutations in exon 21, and insertions in exon 19, or complex mutations).

### Statistical analysis

Clinical, pathological, and molecular variables collected at baseline were summarized as follows: quantitative variables were described using means and standard deviations (SDs), while qualitative variables were represented as percentages. To explore the relationships between mutational status and tumor characteristics, we subjected categorical variables to analysis using either the χ2-test or Fisher’s exact test. Statistical significance was defined as a p-value below 0.05.

The analysis of overall survival in relation to *EGFR* status was conducted employing the Kaplan-Meier method for survival probability estimation, along with assessment of survival differences through the log-rank test, with statistical significance denoted by P<0.05. To address potential confounding factors, survival analysis was initially performed considering various parameters such as age, sex, smoking status, etc. However, no significant associations were observed between these variables and overall survival. Consequently, given the absence of significant associations, Cox proportional hazards regression analysis was not performed to manage potential confounders in the survival analysis. Statistical analyses were performed using IBM SPSS Statistics version 21. Overall survival (OS), assessed as a prognostic marker, was defined as the duration between the date of diagnosis and the date of last follow-up.

## Results

### Patients’ characteristics

A cohort of 302 patients diagnosed with lung cancer was enrolled in this study. A comprehensive summary of the demographic and clinical profiles of the patients is presented in Table 1. The mean age of the patients was 60.34 years, with a SD of 10.80. Gender distribution exhibited a notable male predominance, with males accounting for (N = 220/302, 72.8%) of the cohort. Smoking status was categorized into current or former smokers (N = 164/275, 59.6%) and never-smokers (N = 111/275, 40.4%). Histopathological analysis revealed that adenocarcinoma was the predominant histological subtype, comprising (N = 280/302, 92.7%) of cases. Among adenocarcinoma cases, the acinar subtype was the most prevalent (N = 110/196, 56.1%), followed by solid (N = 86/196, 43.9%), papillary (N = 50/196, 25.5%), mucinous (N = 7/196, 3.6%), lepidic and micropapillary (N = 6/196, 3.10%), with the enteric subtype accounting for only (N = 2/196, 1%). Disease staging indicated that Stage IV tumors were the most frequently diagnosed in the cohort (N = 239/261, 91.5%), whereas Stage IIIA or IIB represented (N = 8/261, 3%) of cases, Stage IA/IB, and IIA/IIB accounted for (N = 1/261, 0,3%), and (N = 4/261, 1,5%) respectively. Metastatic involvement was observed primarily in the lung (N = 110/254, 43.3%), followed by bone metastases (N = 72/254, 28.3%) and lymph node metastases (N = 64/254, 25.1%). 36 patients underwent *EGFR* testing at our hospital but were treated at another institution. The mean follow-up duration for overall survival (OS) was 7.70 months. In terms of molecular characteristics, *EGFR* mutation status was positive for 69 cases (N = 69/302, 22.8%). Interestingly, a double mutation in two different exons was identified in two patients, resulting in a total of 71 *EGFR* mutations among the cohort of 302 patients (N = 71/302, 23.5%).

**Table 1:** Demographic and clinical characteristics of lung cancer patients.

Features	Total
**Age**	
Mean(±SD)	60,34 ± 10,80
<60	119 (44,7%)
>60	147 (55,3%)
**Gender**	
Male	220 (72,8%)
Female	82 (27,2%)
**Smoking status**	
Current or former smoker	164 (59,6%)
Never-smoker	111 (40,4%)
**Histology**	
adenocarcinoma	280 (92,7%)
Squamous	15 (5%)
NOS	5 (1,7%)
Neuroendocrine	1 (0,3%)
Adenosquamous	1 (0,3%)
**Adenocarcinoma subtypes**	
Acinar	110 (56,1%)
Papillary	50 (25,5%)
Micropapillary	6 (3,1%)
Solid	86 (43,9%)
Lepidic	6 (3,1%)
Mucinous	7 (3,6%)
Enteric	2 (1%)
**Mixed tumor**	
Acinar + Papillary	34 (17,3%)
Acinar + Solid	30 (15,3%)
Papillary + Solid	4 (2%)
**Disease stage**	
IA/IB	1 (0,3%)
IIA/IIB	4 (1,5%)
IIIA or IIIB	8 (3%)
IV	239 (91,5%)
**Metastatic sites**	
Lymph node metastasis	64 (25,1%)
Brain	56 (22%)
Pleural	45 (17,7%)
Lung	110 (43,3%)
Liver	51 (20%)
Adrenal	48 (18,8%)
Bone	72 (28,3%)
Skin	5 (1,9%)
***EGFR* mutation**	
Presence	71 (23,5%)
Absence	231 (76,5%)
**Follow-up time (months)**	
Mean (SD)	7,70 (10,42)
Range (month)	1–90

### Mutation distribution

**Fig 1:**
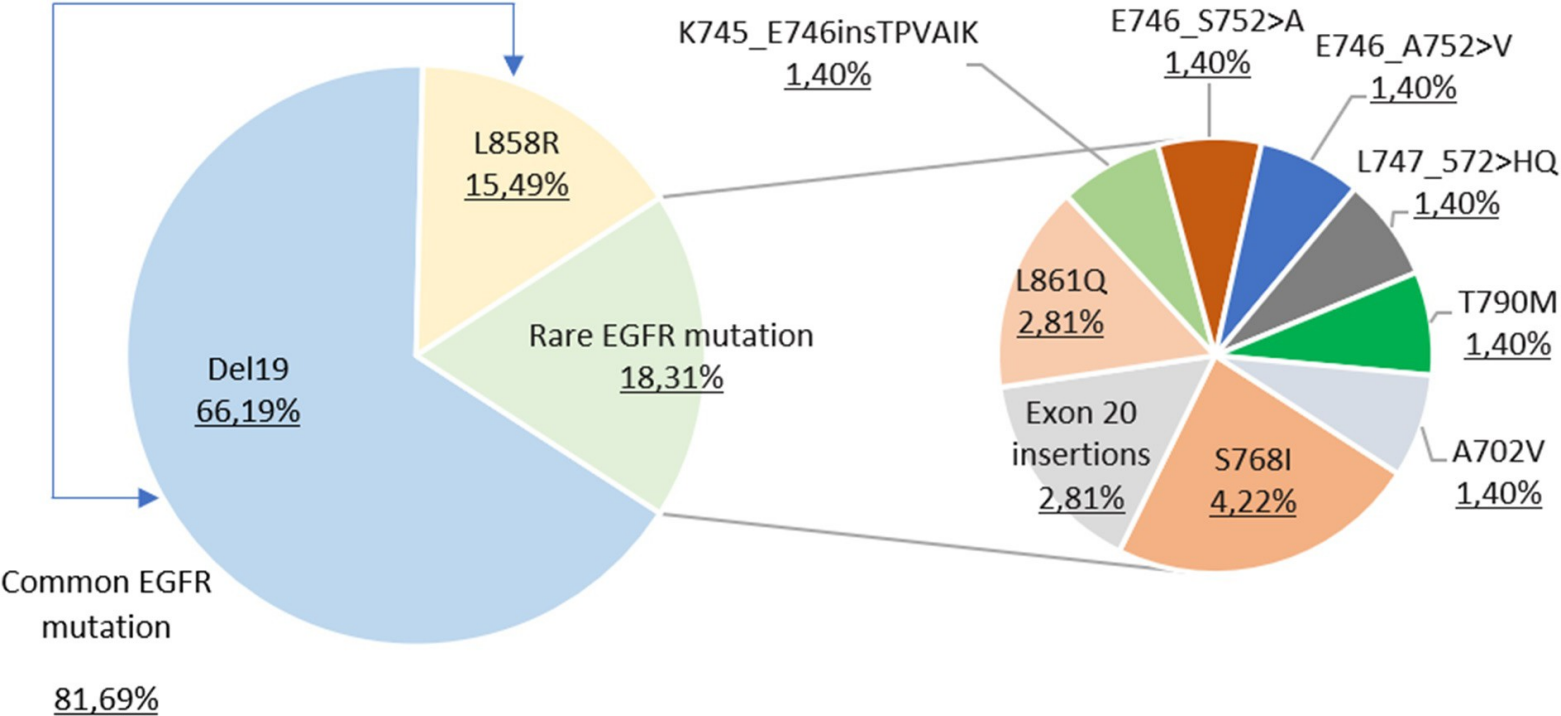
Distribution of *EGFR* mutations. Fig 1 illustrates the prevalence of EGFR mutation subtypes within the study cohort. Deletion 19 (Del19) emerges as the most common mutation, constituting 66.19% of the observed cases, with the L858R point mutation comprising 15.49%. An additional inset highlights the collective frequency of rare EGFR mutations, which altogether make up 18.31%, underscoring the mutation spectrum’s diversity. Among these, the S768I mutation is particularly noteworthy, accounting for 4.22% of the cases with mutations.

The predominant mutational subtype within the spectrum of EGFR mutations was exon 19 deletions (66.19%), followed by the notable presence of the L858R mutation in exon 21 (15.49%). Together, deletions of exon 19 and the L858R mutation, widely recognized as common mutations in the EGFR gene, contribute 81.69% of all mutations detected. Conversely, rare mutations include exon 18 mutation, exon 19 insertional deletions (delins), exon 20 insertions and point mutations such as S768I, T790M in exon 20 and L861Q in exon 21, accounting for 18.3% of the total mutation spectrum (Fig 1).

### Correlation between *EGFR* status and patients’ characteristics

Table 2 presents a comprehensive analysis of the association between clinical and demographic factors and the *EGFR* mutation status within a meticulously studied cohort of lung cancer patients. Age stratification reveals no statistically significant difference in *EGFR* mutation status prevalence between patients aged less than 60 (39.1%) and those aged 60 or older (60.9%) (P = 0.1). Analysis based on gender and smoking status indicates a significant association between *EGFR* mutations and female patients (P = 0.001), as well as a significantly lower percentage of *EGFR* mutations among current or former smokers (32.8%) compared to never-smokers (67.2%) (P < 0.0001). Subtypes of adenocarcinoma exhibit varying associations, with the presence of acinar and, papillary components significantly associated with a higher percentage of *EGFR* mutations compared to tumors without these patterns (P = 0.03) and (P = 0.002) respectively, while a negative association was observed with the solid subtype (P<0,0001). Follow-up time analysis highlights a significantly longer mean follow-up duration among patients with *EGFR* mutations (68.28 months) compared to those with *EGFR* wild-type status (25.79 months) (P = 0.02).

**Table 2:** Association of clinical and demographic factors with *EGFR* mutation status.

Features	Positive *EGFR* mutation	*EGFR* Wild-Type	P value
**Age**			
<60	27 (39,1%)	110 (47,2%)	P = 0,1
>60	42 (60,9%)	123 (52,8%)	
**Gender**			
Female	30 (43,5%)	52 (22,3%)	P = 0,001
Male	39 (56,5%)	181 (77,7%)	
**Smoking status**			
Current or former smoker	21 (32,8%)	143 (67,8%)	P<0,0001
Never-smoker	43 (67,2%)	68 (32,2%)	
**Adenocarcinoma subtypes**			
Acinar			
With	28 (70,0%)	82 (52,6%)	P = 0,03
Without	12 (30,0%)	74 (47,4%)	
Papillary			
With	18 (45,0%)	32(20,5%)	P = 0,002
Without	22 (55,0%)	124 (79,5%)	
Solid			
With	5 (12,5%)	81 (51,9%)	P<0,0001
Without	35 (87,5%)	75 (48,1%)	
**Mixed tumor**			
Acinar + Papillary			
With	10 (25,0%)	24 (15,4%)	P = 0,1
Without	30 (75,0%)	132 (84,6%)	
Acinar + Solid			
With	2 (5,0%)	28 (17,9%)	P = 0,02
Without	38 (95,0%)	128 (82,1%)	
Papillary + Solid			
With	2 (5,0%)	2 (1,3%)	P = 0,1
Without	38 (95,0%)	154 (98,7%)	
**Metastatic sites**			
Lymph node metastasis	12 (21,4%)	52 (26,5%)	
Brain	13 (23,2%)	43 (21,9%)	
Pleural	8 (14,3%)	37 (18,7%)	
Lung	24 (42,9%)	86 (43,9%)	
Liver	13 (22,8%)	38 (19,3%)	
Adrenal	9 (15,8%)	39 (19,9%)	
Bone	21 (37,5%)	51 (26,0%)	P = 0,06
**Follow-up time (months)**			
Mean	68,28	25,79	P = 0,02
SD	6,05	2,19	

### Relationship between *EGFR* mutation subtypes and clinicopathological parameters

Secondly, we conducted a comprehensive analysis of the relationship between common and rare *EGFR* mutation subtypes and clinicopathological parameters, as outlined in Table 3. Our findings illuminate significant associations between common *EGFR* mutations and various clinical features. Notably, female gender (P < 0.0001) and never smoking status (P < 0.0001) displayed strong associations with common *EGFR* mutations. Additionally, specific adenocarcinoma subtypes, particularly the papillary subtype, exhibited robust associations with common *EGFR* mutations (P = 0,001). Additionally, Patients with common *EGFR* mutations also had a better overall survival (OS) than patients with wild-type *EGFR* (median OS: 67.85 months vs. 25.79 months; P = 0.04). These results suggest that *EGFR* mutation subtype is an important prognostic factor in patients with advanced NSCLC.

**Table 3:** Association between *EGFR* mutation subtypes (common, rare, and WT) and, clinicopathological factors.

Features	Common *EGFR* mutation	*EGFR* Wild-Type	P value	Rare *EGFR* mutation	*EGFR* Wild-Type	P value
**Age**						
<60	39,3%	47,2%	P = 0,1	38,5%	47,2%	P = 0,3
>60	60,7%	52,8%		61,5%	52,8%	
**Gender**						
Female	46,4%	22,3%	P<0,0001	30,8%	22,3%	P = 0,3
Male	53,6%	77,7%		69,2%	77,7%	
**Smoking status**						
Current or former smoker	26,4%	67,8%	P<0,0001	63,6%	67,8%	P = 0,5
Never-smoker	73,6%	32,2%		36,4%	32,2%	
**Adenocarcinoma subtypes**						
Acinar						
With	67,6%	52,6%	P = 0,07	83,3%	52,6%	P = 0,1
Without	32,4%	47,4%		16,7%	47,4%	
Papillary						
With	50,0%	20,5%	P = 0,001	16,7%	20,5%	P = 0,6
Without	50,0%	79,5%		83,3%	79,5%	
Solid						
With	11,8%	51,9%	P<0,0001	16,7%	51,9%	P = 0,09
Without	88,2%	48,1%		83,3%	48,1%	
**Mixed tumor**						
Acinar + Papillary						
With	26,5%	15,4%	P = 0,1	16,7%	15,4%	P = 0,6
Without	73,5%	84,6%		83,3%	84,6%	
Acinar + Solid						
With	2,9%	17,9%	P = 0,01	16,7%	17,9%	P = 0,7
Without	97,1%	82,1%		83,3%	82,1%	
Papillary + Solid						
With	5,9%	1,3%	P = 0,1	0,0%	1,3%	P = 0,7
Without	94,1%	98,7%		100,0%	98,7%	
**Metastatic sites**						
Lymph node metastasis	17,8%	26,5%	P = 0,1	36,4%	26,5%	P = 0,1
Brain	24,4%	21,9%		18,2%	21,9%	
Pleural	11,1%	18,7%		27,3%	18,7%	
Lung	40,0%	43,9%		54,5%	43,9%	
Liver	22,2%	19,3%		25,0%	19,3%	
Adrenal	13,3%	19,9%		25,0%	19,9%	
Bone	35,6%	26,0%		45,5%	26,0%	
**Follow-up time (months)**						
Mean	67,85	25,79	P = 0,04	42,91	25,79	P = 0,3
SD	6,81	2,19		7,60	2,19	

Table 4 illustrates the comparison of clinicopathological characteristics between common and rare mutations. No statistically significant differences were revealed between the distribution of age, gender, adenocarcinoma subtypes, metastatic sites, or follow-up time between patients with common *EGFR* mutations and patients with rare *EGFR* mutations. However, there was a trend towards younger age (38.5% vs. 39.3%, P = 0.1), male gender (69.2% vs. 53.6%, P = 0.2), and metastatic disease to the brain and pleural (18.2% and 27.3% vs. 24.4% and 11.1%, respectively, P = 0.4 for both comparisons) in patients with rare *EGFR* mutations. Additionally, patients with rare *EGFR* mutations were more likely to be current or former smokers than patients with common EGFR mutations (63.6% vs. 26.4%, P = 0.01).

**Table 4:** Clinicopathological characteristics stratified by common and rare *EGFR* mutation subtypes.

Features	Common *EGFR* mutation	Rare *EGFR* mutation	P value
**Age**			
<60	39,3%	38,5%	P = 0,1
>60	60,7%	61,5%	
**Gender**			
Female	46,4%	30,8%	P = 0,2
Male	53,6%	69,2%	
**Smoking status (n = 257)**			
Current or former smoker	26,4%	63,6%	P = 0,01
Never-smoker	73,6%	36,4%	
**Adenocarcinoma subtypes (n = 196)**			
Acinar			
With	67,6%	83,3%	P = 0,4
Without	32,4%	16,7%	
Papillary			
With	50,0%	16,7%	P = 0,1
Without	50,0%	83,3%	
Solid			
With	11,8%	16,7%	P = 0,5
Without	88,2%	83,3%	
**Mixed tumor**			
Acinar + Papillary			
With	26,5%	16,7%	P = 0,5
Without	73,5%	83,3%	
Acinar + Solid			
With	2,9%	16,7%	P = 0,2
Without	97,1%	83,3%	
Papillary + Solid			
With	5,9%	0,0%	P = 0,5
Without	94,1%	100,0%	
**Metastatic sites (n = 254)**			
Lymph node metastasis	17,8%	36,4%	P = 0,1
Brain	24,4%	18,2%	
Pleural	11,1%	27,3%	
Lung	40,0%	54,5%	
Liver	22,2%	25,0%	
Adrenal	13,3%	25,0%	
Bone	35,6%	45,5%	
**Follow-up time (months)**			
Mean	67,85	42,91	P = 0,9
SD	6,81	7,60	

### Prognostic impact of *EGFR* mutation subtypes on overall survival in NSCLC: A comparative analysis

**Fig 2:**
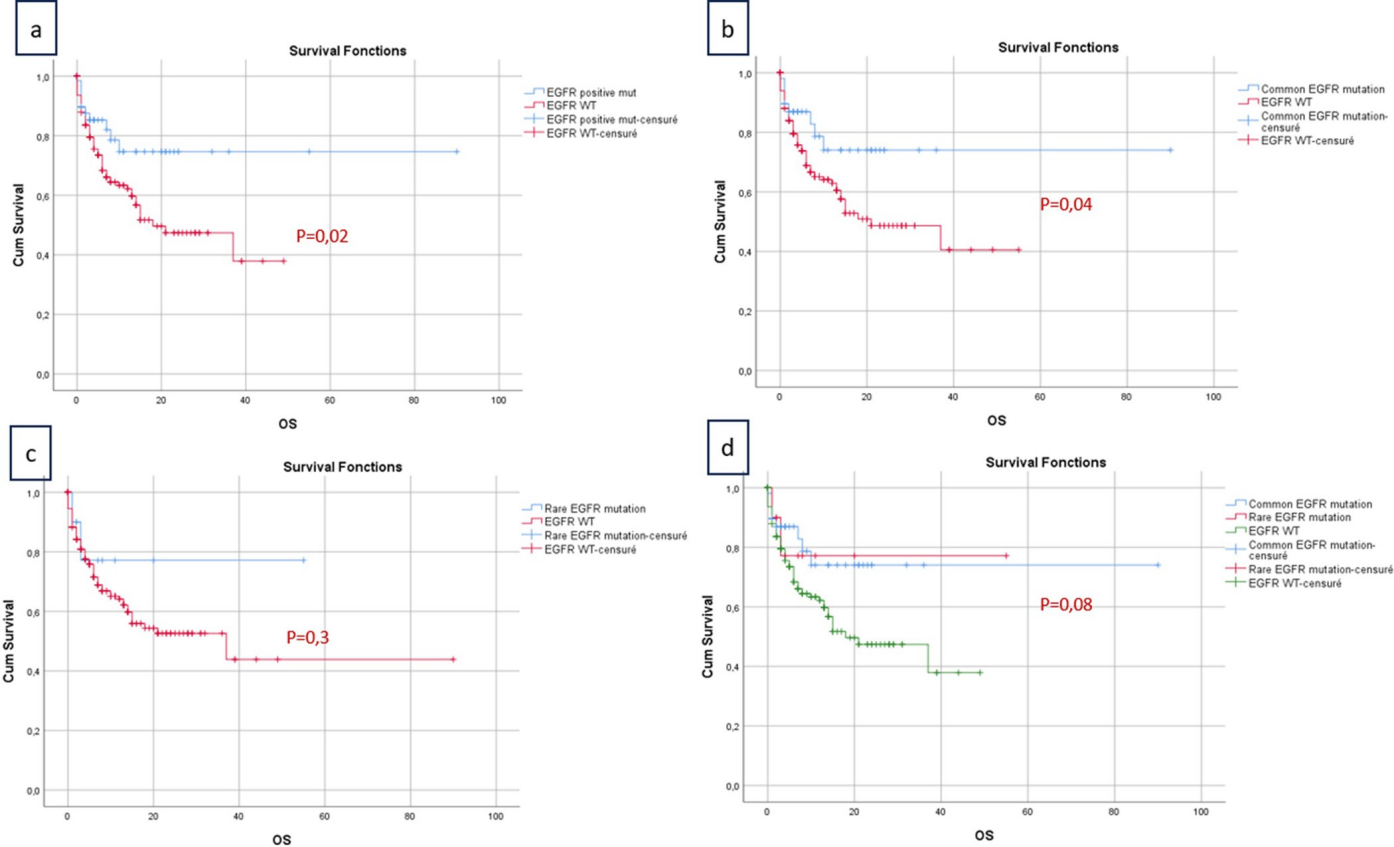
Analysis of overall survival in different *EGFR* subgroups. (a) shows overall survival for patients with EGFR mutations compared to wild-type, with a clear survival benefit for those with mutations (p = 0.02). (b) compares survival between common EGFR mutations and wild-type, with a notable difference (p = 0.04). (c) displays survival for rare EGFR mutations versus wild-type, showing no significant difference (p = 0.3). (d) compares common and rare EGFR mutations against wild-type, with a slight but not significant survival improvement in common mutations (p = 0.08).

The analysis of the prognostic implications associated with *EGFR* mutation status unveiled a significant correlation between the presence of *EGFR* mutations in patients treated with TKIs and overall survival. Notably, the group characterized by EGFR mutations exhibited a notably higher overall survival, with a mean of 68.28 months (95% CI: 56.41% - 80.15%; p = 0.02). Moreover, in the comparative analysis of the three distinct groups (common, rare, and wild-type), the p-value demonstrated a notable proximity to statistical significance (p = 0.08). It was evident that patients with common *EGFR* mutations treated with TKIs exhibited the most favorable overall survival, characterized by a mean of 67.85 months (95% CI: 54.49% - 81.22%). Subsequently, patients within the group with rare *EGFR* mutations demonstrated a notably lower mean overall survival of 42.91 months (95% CI: 28.00% - 57.82%), while the EGFR wild-type group exhibited the least mean overall survival, measuring 25.79 months (95% CI: 21.48% - 30.09%).These observations underscore the prognostic significance of specific EGFR mutation subtypes and bear relevance for the development of tailored therapeutic strategies within the context of non-small-cell lung cancer (Fig 2). No significant differences in survival were identified concerning other factors such as age, gender, smoking history, histological type, and disease stage.

## Discussion

*EGFR* mutations and the *EGFR* tyrosine kinase inhibitors play a pivotal role in the landscape of non-small cell lung cancer research. *EGFR* mutations have received significant attention due to their therapeutic and prognostic implications, particularly with the advent of *EGFR* TKIs. These mutations have been extensively studied because of their importance in determining treatment selection and patient outcomes [23]. To the best of our knowledge, this study is among the first to investigate the prevalence and clinical and pathological significance of *EGFR* mutation subtypes in patients with advanced NSCLC in the Moroccan population and the Middle East and North Africa region, as well as to assess the prognostic implications of *EGFR* mutation subtypes in these patients.

This study examines a patient cohort who underwent *EGFR* testing at a Moroccan healthcare center. Screening 302 lung cancer patients identifies 71 cases (23.5%) with *EGFR* mutations, including 13 instances of rare and complex *EGFR* mutations. Notably, patients with these rare or complex *EGFR* mutations exhibit a statistically significant association with a history of smoking, in contrast to patients bearing classic *EGFR* mutations. Furthermore, disparities in overall survival (OS) outcomes emerge when compared to patients with common *EGFR* mutations. A substantial proportion of patients with common *EGFR* mutations receive *EGFR* Tyrosine Kinase Inhibitors as first or second-line treatment, whereas cases with rare mutations benefit from targeted therapy in only two cases, specifically those with the L858R+S768I double mutation and the S768I mutation. OS analysis reveals that patients with common *EGFR* mutations who receive TKI treatment experience the longest OS, diverging from cases with rare *EGFR* mutations and those with wild-type *EGFR* status.

In recent years, the treatment paradigm for lung cancer has undergone a significant transformation, transitioning from a one-size-fits-all platinum-based chemotherapy regimen to a more individualized and targeted approach. This personalized treatment strategy necessitates a more refined characterization of tumor features, encompassing precise histological subclassifications, tumor typing, and comprehensive molecular pathological testing. In particular, for adenocarcinomas, it has become customary to perform *EGFR* mutation testing and rearrangements involving the *ALK* and *ROS1* genes screening [24]. In previous randomized clinical trials, the efficacy of EGFR TKIs in enhancing survival rates, extending lifespan, and improving quality of life has been demonstrated in patients with *EGFR*-mutated advanced NSCLC [25, 26].

In this study, we aimed to compare the clinicopathological characteristics and overall survival of patients with distinct *EGFR* mutation statuses (rare, common, and wild-type) by conducting a prospective analysis of a cohort of non-small cell lung cancer patients who underwent *EGFR* testing at a Moroccan healthcare facility.

In the present cohort, a substantial 23.5% of patients demonstrated *EGFR* mutations. The majority of these mutations were of the common subtype, comprising 81.69% of cases, while 18.31% of patients exhibited rare *EGFR* mutations. It is noteworthy that our analysis discloses a statistically significant prevalence of common *EGFR* mutations among women and non-smokers within the cohort. These findings concur with previous European, American, and Asian investigations [1, 27–30], which have consistently demonstrated a comparable distribution of *EGFR* mutations across various cohorts.

Further investigation has unveiled a significant association between smoking habits and the specific subtypes of *EGFR* mutations. Existing literature reports that patients harboring common *EGFR* mutations are less likely to have a history of current or former smoking [31]. Compared to patients with classic mutations, our data indicate that patients with rare or complex mutations have a significantly higher positive smoking status. These observations align with recent studies that have similarly documented an increased prevalence of former or current smokers in the rare or complex mutation subgroup [1, 32]. In a study conducted by Lohinai et al. [33], a significant correlation was observed between rare EGFR mutations and smoking status. Within their cohort, 37 out of 49 cases with rare mutations were identified as smokers. This results aligns with findings from two other European studies involving cohorts of 23 and 14 patients with rare mutations, respectively [1, 27]. However, investigations conducted within Asian populations did not support that smoking exerts a significant influence on the emergence of complex or infrequent *EGFR* mutations [34, 35]. Our findings suggest that a positive smoking status may raise the risk of uncommon or complex *EGFR* mutations in Moroccan patients.

Within our study cohort, patients harboring *EGFR* mutations and treated with EGFR tyrosine kinase inhibitors exhibited a significantly better prognosis when compared to patients with wild-type *EGFR* status who received chemotherapy. A congruent outcome was observed in a larger cohort comprising 3,062 patients diagnosed with metastatic non-small-cell lung cancer [36]. The median overall survival within the *EGFR*-mutated subgroup surpassed that of the wild-type *EGFR* cohort. Similar findings were reported in a distinct study encompassing a Caucasian population of 285 non-small-cell lung cancer patients, wherein patients with *EGFR* mutations demonstrated a markedly prolonged overall survival compared to their wild-type *EGFR* counterparts [37]. Furthermore, within an Asian patient cohort of 424 cases, Zheng et al. reported that patients with *EGFR* mutations experienced a significantly longer survival duration to those with wild-type *EGFR* [38]. Additionally, an American study involving 1036 cases further corroborates these findings, revealing that patients with EGFR-mutated tumors treated with TKIs exhibited a notably prolonged overall survival (OS), with a median duration of 34 months (95% CI, 32–39). Conversely, those with tumors wild-type for EGFR showed a comparatively shorter median OS of 23 months (95% CI, 20–26) [39].

The comparison of overall survival between patients with common *EGFR* mutations and rare *EGFR* mutations provides valuable insights into the clinical implications of these distinct mutation profiles in non-small-cell lung cancer. Our findings reveal a noteworthy difference in mean overall survival, with patients harboring common *EGFR* mutations exhibiting a significantly longer mean survival of 67.85 months. This observation aligns with Asian American, and European literature, where common *EGFR* mutations, such as exon 19 deletions and L858R point mutations, have been consistently associated with improved clinical outcomes, including longer progression-free survival and overall survival, when treated with *EGFR* tyrosine kinase inhibitors like gefitinib and erlotinib [40–42]. These drugs have demonstrated high response rates in patients with common mutations, owing to their enhanced sensitivity to targeted therapy.

Conversely, patients with rare *EGFR* mutations in our dataset exhibit a notably shorter mean overall survival of 42.91 months, a pattern that corresponds with studies highlighting the clinical challenges associated with these mutations [43, 44]. Rare *EGFR* mutations encompass a heterogeneous group of alterations, often less responsive to standard *EGFR* TKIs and requiring alternative treatment approaches [15, 45]. However, the observed difference in overall survival between rare *EGFR* mutations and wild-type *EGFR* did not reach statistical significance in our analysis (P = 0.3), potentially reflecting the limited sample size or the diversity within the rare mutation category.

The occurrence of multiple mutations within the EGFR gene is a rare phenomenon [46]. Castañeda-González et al,. [47] conducted a systematic review comprising 41 articles, revealing that 0.95% of the patients included in the studies exhibited multiple mutations in the EGFR gene. Notably, the exon 20 mutation (T790M) emerged as the predominant single mutation associated with dual mutations. Within the subset of patients harboring the T790M mutation, a higher frequency of acquired T790M mutations was observed in those characterized by a deletion in exon 19, while de novo mutations were more prevalent in patients with the L858R mutation. In our study, we identified two cases with multiple EGFR mutations, specifically L858R+S768I and L858R+de novo T790M. This observation aligns with the findings reported by Castañeda-González et al., where de novo T790M was combined with L858R [47]. However, the clinical significance of these compound mutations, comprising 2.89% of the EGFR-mutated cases in our cohort, necessitates nuanced consideration. While the number of instances with multiple mutations in our study is limited, their presence is consistent with broader cohorts reporting the existence of complex mutational profiles [48, 49]. These intricate mutations may introduce additional challenges in treatment selection and response assessment.

This study has several limitations. Firstly, the relatively modest sample size may constrain the statistical power to identify significant differences in overall survival between rare and common EGFR mutations. The single-center nature of the study may limit the direct generalizability of the findings to other populations, necessitating larger, multicenter studies for confirmation and exploration of clinical implications across diverse cohorts. The limited sample size within the rare mutation category could impact the detection of statistically significant differences. Future investigations should prioritize larger multicenter studies with diverse populations, enhancing the comprehensive understanding of clinical implications associated with EGFR mutation subtypes. Longitudinal studies with extended follow-up periods are recommended to explore the long-term effects of EGFR mutations on patient outcomes and provide a nuanced assessment of prognosis. To address potential confounding factors, comprehensive adjustments, including age, sex, smoking history, and other clinical parameters, should be incorporated in future research. Additionally, investigating the influence of specific treatments, such as EGFR tyrosine kinase inhibitors, on survival outcomes is crucial for a nuanced interpretation of the association between EGFR mutations and overall survival.

## Conclusion

Our study underscores the importance of *EGFR* mutation testing in non-small cell lung cancer patients, particularly in guiding treatment decisions. The associations between *EGFR* mutation subtypes and clinical characteristics, such as non-smoking history and female gender with common mutation, and positive smoking history with rare mutation, emphasize the need for tailored approaches to treatment. The significantly improved overall survival of patients with common *EGFR* mutations highlights the clinical relevance of identifying and targeting these mutations with *EGFR* TKIs. However, the study also reveals the challenges associated with rare *EGFR* mutations, which may require alternative treatment strategies to improve patient outcomes.

## Supporting information

S1 Data (XLSX)
